# The Contribution of Temporal Flat Lateral Position on the Mortality and Discharge Rates of Older Patients with Severe Dysphagia

**DOI:** 10.3390/ijerph18168443

**Published:** 2021-08-10

**Authors:** Akiko Shimizu, Ryuichi Ohta, Hana Otani, Chiaki Sano

**Affiliations:** 1 Department of Nursing, Unnan City Hospital, 96-1 Iida, Daito-cho, Unnan City 699-1221, Shimane, Japan; aki6ika@gmail.com; 2 Community Care, Unnan City Hospital, 96-1 Iida, Daito-cho, Unnan City 699-1221, Shimane, Japan; 3 Department of Rehabilitation, Unnan City Hospital, 96-1 Iida, Daito-cho, Unnan City 699-1221, Shimane, Japan; blossom0409@gmail.com; 4 Department of Community Medicine Management, Faculty of Medicine, Shimane University, 89-1 Enya cho, Izumo 693-8501, Shimane, Japan; sanochi@med.shimane-u.ac.jp

**Keywords:** dysphagia, temporal flat lateral position, TFLP, older adults, rehabilitation medicine

## Abstract

Severe dysphagia leads to mortality in older patients and hinders their discharge from hospitals. The temporal flat lateral position (TFLP) enables them to continuously eat, thus resolving the aforementioned issues. We aimed to explore the effect of TFLP on the mortality and discharge rates of older patients with severe dysphagia. This interventional study comprised a historical control of patients admitted to a rural community hospital from January 2019 to December 2020 and diagnosed with severe dysphagia. The primary outcomes included the mortality and the rate of discharge from the hospital. While the intervention group was treated with TFLP, the control group underwent no treatment. We selected 79 participants (intervention group = 26, control group = 53), with an average age of 87.9 years. The discharge rate was significantly higher in the intervention group than in the control group (57.7% vs. 26.4%, *p* = 0.012). The mortality rate was also significantly lower in the intervention group compared to the control group (34.6% vs. 71.7%, *p* = 0.003). TFLP can improve the discharge and mortality rates in community hospitals, thereby improving patient outcomes. Clinicians should focus on practical education and the implementation of TFLP in communities in order to promote it.

## 1. Introduction

Dysphagia has been identified as a critical problem underlying the health conditions of older people in aging societies. Dysphagia can be caused by various diseases such as infections, heart failure, dementia, and disuse syndrome [1,2,3]. Aging can cause a deterioration of physical and psychological abilities, thus leading to the progression of disuse syndrome [4]. The deterioration impinged on older people in homes can be associated with the difficulty in the continuity of home care and worsening mortality rates [5,6,7]. In Japan, a country with one of the most elderly populations in the world, high mortality diseases such as pneumonia and aspiration pneumonia have become more prevalent, following cancer, heart diseases, and cerebrovascular diseases [8]. Previous studies have shown that 70% of the patients with pneumonia are >75 years old and are diagnosed with aspiration pneumonia [2]. Furthermore, more than 20% of the patients aged > 75 years old and with dysphagia die after hospital admission [2]. In addition, 50% of the patients with pneumonia and dysphagia die within 1 year [9]. Thus, dysphagia is significantly related to the mortality of older patients, thereby making it necessary to sustain and improve their ability to swallow through appropriate rehabilitation. In contrast, the progression of dysphagia can be triggered by various factors. Therefore, implementing interventions to address the problem of dysphagia in aging societies can be difficult.

Older patients with dysphagia require multiple interventions because the condition is caused by various factors such as cerebrovascular diseases, neurogenic diseases, and sarcopenia. Owing to the decline in cognitive function, swallowing training can be challenging [10,11,12]. Swallowing can be classified into the following five phases: recognition, oral preparatory, oral transport, pharyngeal, and esophageal [13]. Appropriate treatment and rehabilitation can be performed by judging the impaired phase [3]. Aging can weaken the pharyngeal muscles, which can severely influence swallowing. Thus, reclining positions can increase the risk of food entering the larynx and the risk of aspiration [11,14]. The progression of dysphagia can critically deter the continuity of home care, which, in turn, can increase the duration of admission and the exhaustion of hospital medicine [15,16,17]. The ability to swallow can improve the possibility of longer and qualified home care for older people with dysphagia [3]. Moreover, swallowing methods that do not employ a reclining position would enable such patients to sustain swallowing. Therefore, to improve the quality of life of older patients with severe dysphagia, new swallowing methods should be developed.

**Figure 1:**
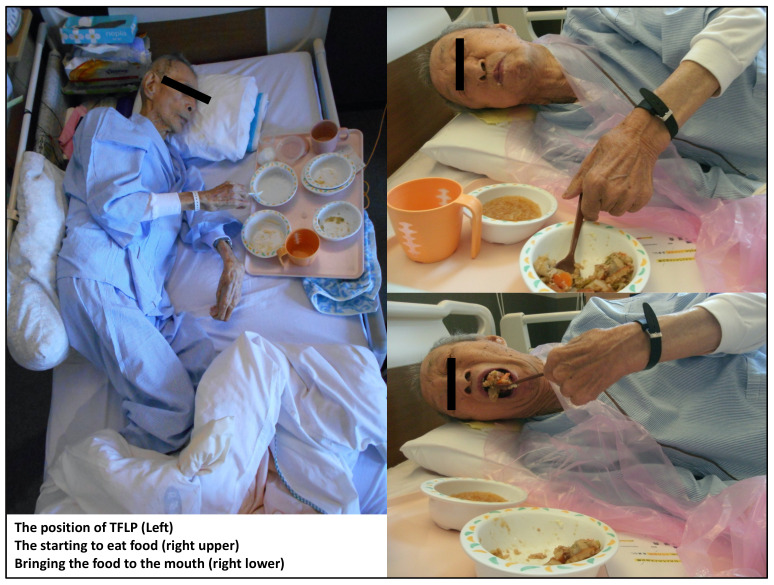
The process of temporal flat lateral position. TFLP; temporal flat lateral position.

The temporal flat lateral position (TFLP) was developed for older patients with dyspnea who cannot consume food in the usual ways. TFLP can widen the space for food collection in the pharynx, thus preventing aspiration while eating [18]. The usual space for collecting food in the pharynx is approximately 3 cc. Patients with dysphagia typically have pharyngeal muscle dysfunction. In such cases, the usual eating position can allow the mass of food to enter the pharynx, with the effect of gravity causing aspiration. In contrast, TFLP can widen the space to 15–20 cc without the influence of gravity, thereby reducing the risk of aspiration (Figure 1). By using TFLP, older patients with severe dysphasia and those who cannot eat food in conventional ways may be able to eat. Thus, TFLP may enable such patients admitted to hospitals to live longer and be discharged to their homes or other medical facilities. However, no study has investigated the effect of TFLP on the mortality and discharge rates of older patients with severe dysphagia. Therefore, we aimed to clarify the impact of TFLP on the mortality and discharge rates of older patients who had been admitted to a rural community hospital and diagnosed with severe dysphagia and the inability to eat in conventional ways.

## 2. Materials and Methods

### 2.1. Setting

Unnan City is a rural city located southeast of Shimane Prefecture, Japan. In 2020, the total population was 37,638 (18,145 men and 19,492 women). Older individuals (aged > 65 years) account for 39% of the population, and this proportion is projected to increase to 50% by 2025 [19]. Unnan City Hospital was the only public hospital during the study. The hospital staff comprised 27 physicians, 197 nurses, 7 pharmacists, 15 clinical technicians, 37 therapists (22, 12, and 3 physical, occupational, and speech therapists, respectively), 4 nutritionists, and 34 clerks. Unnan City only has one recovery rehabilitation unit [20].

### 2.2. Participants

We performed a quasi-experimental study; all patients were aged > 65 years, with severe dysphagia, and had been admitted to the Unnan City Hospital between 1 January 2019 and 31 December 2020. The nurses assessed all patients for their ability to swallow using a water swallowing test (WST) in order to screen for dysphagia [21]. After obtaining positive WST results, the patients were assessed using a videoendoscopic test (VE) by an otorhinolaryngologist for the risk of dysphagia [22]. Upon observation of a delay in swallowing or a swallowing dysfunction with no confirmed aspiration, the patients were diagnosed with dysphagia and rehabilitated by speech therapists. If aspiration was confirmed, they underwent a video fluoroscopic examination (VF) for the appropriate suggestion of food [23]. Dysphagia was defined based on the diagnosis of an otorhinolaryngologist. The criteria for severe dysphagia were as follows: (i) <3 points of WST in any reclining position at bedside or (ii) the presence of aspiration or food entry into the larynx in VE or VF [21]. TFLP began in June 2019 in patients with severe dysphagia as confirmed by the WST, following an agreement by the patient or their family. Starting in April 2020, TFLP was performed in patients with severe dysphagia as confirmed by WST, VE, or VF. A speech therapist and a nurse specializing in dysphagia nursing, who had been trained in TFLP in May 2019, performed the intervention. They were charged with the assessment of the swallowing function, for 2 days every week. The intervention group was defined as the patients with severe dysphagia who were trained to feed in the TFLP. The control group was defined as the patients aside from those in the intervention group.

### 2.3. Measurements

Patient information was extracted from the electronic medical records of the Unnan City Hospital. The main outcome was readmission to the hospital. We recorded the age, sex, body mass index, and serum albumin (g/dL) as indicators of the nutritional status; the Charlson Comorbidity Index (CCI) calculated from the medical histories, with the score demonstrating the severity of the patient’s medical conditions [24]; and the care level based on the Japanese long-term insurance system (numbered from 1 to 5, with 1 denoting least dependent, 5 denoting severely dependent), which was determined by primary care physicians’ assessments and discussions with multiple healthcare professionals [25], the cognitive and motor components of the Functional Independence Measure (FIM) at admission—measured by the therapists as an indicator of the patients’ ADL, and the mortality and discharge rates from the hospital.

### 2.4. Analysis

To analyze the differences in participant characteristics and the mortality and discharge rates between the two groups, we performed *t*-tests and χ^2^ tests. A significance level of *p* < 0.05 was used for all comparisons. At least 20 participants were required in each group based on α = 0.05, β = 0.10, and power of 90%. Moreover, a between-group difference of 40% was required in relation to mortality. We excluded cases with missing data from the analysis. All statistical analyses were performed using EZR v1.50, a graphical user interface for R (The R Foundation; http://www.r-project.org, accessed on 24 June 2021) [26].

### 2.5. Ethical Consideration

The anonymity and confidentiality of patient information were ensured. We informed the patients and their families about the use of their clinical data for publication and obtained their informed consent. The research protocol was posted on the hospital’s website without any information about the patients. We listed the contact information of the hospital representative responsible for answering any queries about this research. All procedures of this study were performed in compliance with the tenets of the Declaration of Helsinki and its later amendments. The Unnan City Hospital Clinical Ethics Committee approved the study protocol (no. 20200030).

## 3. Results

### 3.1. The Demographics of the Participants

**Figure 2:**
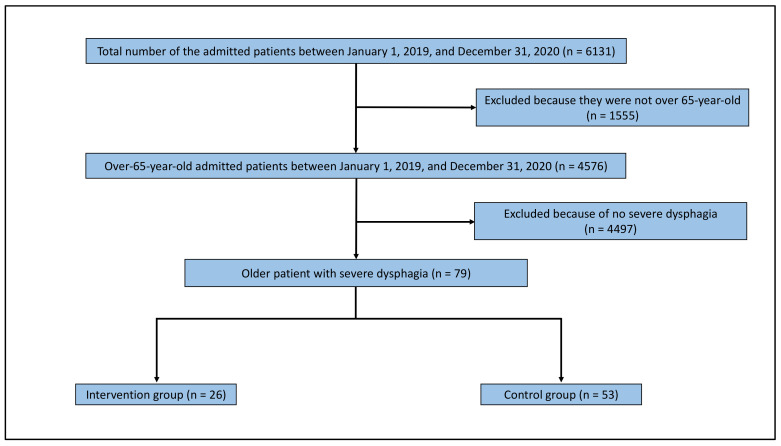
Flow chart of the study population.

Figure 2 depicts the flowchart for selecting the study population. A total of 6131 patients had been admitted to the Unnan City Hospital between 1 January 2019 and 31 December 2020. Of these patients, 4576 were aged over 65 years. Moreover, 79 patients had severe dysphagia. Twenty-six patients were enrolled in the intervention group handled by a speech therapist with TFLP skills and a nurse specialized in dysphagia nursing. The control group comprised 53 patients. Their average age was 87.9 years. The intervention and control groups comprised 69.2% (18/26) and 75.5% (40/53) men, respectively. There were no statistically significant differences in the demographic data between the groups (Table 1).

### 3.2. Differences in the Duration of Hospitalization, Mortality, and Discharge Rates

The duration of hospitalization was significantly longer in the intervention group than in the control group (61.5% vs. 30.0%, *p* = 0.008). The intervention group demonstrated significantly lower mortality than the control group (34.6% vs. 71.7%, *p* = 0.003). The discharge rate was significantly higher in the intervention group than in the control group (57.7% vs. 26.4%, *p* = 0.012) (Table 2).

### 3.3. The Diseases as Reasons for Admission among the Participants

The most frequent reasons for admission were aspiration pneumonia (*n* = 21, 26.58%), bacterial pneumonia (*n* = 17, 21.52%), and urinary tract infection (*n* = 13, 16.47%) (Table 3).

## 4. Discussion

Our findings demonstrated the effects of TFLP on the mortality and discharge rates of older patients with severe dysphagia. TFLP decreased mortality and increased the discharge rate from hospitals. However, it could also prolong the duration of admission. Aging societies will continue to see an increase in the number of older patients with dysphagia suffering from critical diseases such as pneumonia and urinary tract infections. The efficient use of TFLP through dialogue with older patients and their families can be vital for enhancing the quality of life (QoL) of the former.

TFLP enabled the patients with severe dysphagia to eat continuously using their mouth, thereby improving the mortality and discharge rates at community hospitals. Oral intake and swallowing movements involve various muscles of the face and neck, which can stimulate both the muscles and neurological systems [27,28]. The use of the mouth in patients with dysphagia, with or without gastrostomy, improves mortality by sustaining their QoL [29,30,31]. Total parenteral nutrition, gastrostomy, or a natural course without intensive nutritional interventions can be selected for such patients. Natural courses without intensive nutritional interventions can terminate patient lives. However, total parenteral nutrition can deter their gastrointestinal functions and immunological impairments, thus deteriorating their systemic condition and increasing mortality as compared to the use of gastrostomy [32,33]. Gastrostomy is negatively associated with prolonged lifespan worldwide [34,35]. Additionally, in Japan, the negative image of gastrostomy might inhibit its selection by patients and their families [36]. Therefore, TFLP can be a reasonable option to facilitate oral food intake in patients with severe dysphasia. A previous study reported that TFLP improves activities of daily life, and our current pilot study demonstrated the additional value of TFLP in improving the mortality and discharge rates [18]. Our research necessitates subsequent studies to investigate the long-term effects of TFLP with an experimental design.

The practical implementation of TFLP may affect the duration of hospitalization. Despite its effectiveness in patients with severe dysphagia, the performance of TFLP by patients and caregivers is time-consuming; thus, the duration of hospitalization was significantly longer in the TFLP group. Healthcare systems in different counties can control the length of hospitalization [37]. In Japan, patients requiring longer time for recovery can be transferred from acute care units to comprehensive care or rehabilitation units in community hospitals [20]. The duration of hospitalization of older patients in Japan is approximately 120 days [20], and in this study, the length of hospital stay could not be shorter as the eating disability limited the discharge of patients with severe dysphagia. The reacquisition of the patients’ eating ability is important as it may allow them to be discharged and return to their previous situations in their communities, thus ensuring sustainable, comprehensive care [38]. In other countries, patients may have a shorter hospitalization period. Moreover, various situations should be considered in nursing homes and home care while implementing TFLP.

The TFLP can be used in community settings and would require collaboration with various home-based medical professionals such as home care nurses, home care workers, and care managers. Aging societies suffer from various healthcare issues in relation to interprofessional collaboration, particularly in rural contexts [39,40,41]. TFLP-related knowledge and skills of the aforementioned professionals would enable patients with dysphagia to be trained in TFLP in their homes and to eat continuously for the rest of their lives outside hospitals. However, the limitation for TFLP progression includes not only the inadequate knowledge and skills of medical professionals but also the burden on their work in communities and healthcare facilities [28,42]. Future studies should investigate the progression of TFLP by providing information and education in communities while respecting the workload and mental and physical burdens of the health professionals involved. Furthermore, the most frequent reasons for patient admission were pneumonia and UTI, which are common in older people. Therefore, older patients have a high risk of severe dysphagia. Thus, decision-making regarding TFLP usage should be discussed with patients and their families, considering the limitations in their homes. Patients with severe dysphagia have limited longevity. Therefore, a longer duration of admission may lessen their lives outside hospitals and increase the risk of in-hospital death. Hence, the use of TFLP should likewise consider the longevity of patients.

This study had several limitations. First, the absence of a strict inter-group comparison created a sampling bias. Considering the pilot design, subsequent experimental studies are needed to establish valid evidence. Another limitation was the setting of the study. It was performed in a Japanese rural community hospital, such that the demographics could be unique. However, dysphagia is prevalent worldwide, principally in the elderly. Therefore, TFLP can be applied to other settings to confirm its effectiveness in order to establish more valid evidence.

## 5. Conclusions

TFLP improved the discharge rates and mortality in patients admitted to the community hospital, besides enhancing the quality of patient outcomes. This study suggests that community health professionals should consider practical education and the implementation of TFLP in the communities to enhance the quality of life of older people with dysphagia. Subsequent experimental studies should validate our findings regarding the effects of TFLP on the patient discharge rate and mortality as well as investigate the impact of TFLP education in communities on the perceptions of healthcare professionals and patients and health outcomes.

## Figures and Tables

**Table 1:** Patient demographics.

Factor	Intervention (*n* = 26)	Control (*n* = 53)	*p*-Value
Age, mean (SD)	87.04 (8.30)	87.91 (7.64)	0.646
Male sex (%)	18 (69.2)	40 (75.5)	0.595
Albumin, mean (SD)	3.10 (0.49)	3.21 (0.66)	0.471
BMI, mean(SD)	19.11 (3.78)	18.73 (3.25)	0.643
Hemoglobin, g/dL (SD)	11.52 (1.32)	11.93 (1.97)	0.339
Creatinine, mg/dL (SD)	0.85 (0.41)	0.99 (0.76)	0.403
eGFR, mL/min 1.73 m^2^ (SD)	65.26 (20.09)	61.95 (23.04)	0.533
Care level (%)			
0	3 (11.5)	7 (13.2)	0.931
1	2 (7.7)	4 (7.5)	
2	6 (23.1)	9 (17.0)	
3	3 (11.5)	10 (18.9)	
4	7 (26.9)	16 (30.2)	
5	5 (19.2)	7 (13.2)	
Coming from home (%)	13 (50.0)	21 (39.6)	0.47
FIM score at admission			
Cognitive domain	20.08 (10.54)	15.48 (10.89)	0.08
Motor domain	45.92 (30.92)	38.29 (26.40)	0.259
CCI. (%)			
Score = 4	6 (23.1)	7 (13.2)	0.702
Score = 5	9 (34.6)	14 (26.4)	
Score = 6	5 (19.2)	13 (24.5)	
Score = 7	4 (15.4)	9 (17.0)	
Score = 8	1 (3.8)	7 (13.2)	
Score = 9	1 (3.8)	3 (5.7)	
Dementia (%)	9 (34.6)	24 (45.3)	
Diabetes (%)	6 (23.1)	10 (18.9)	
Heart failure (%)	3 (11.5)	9 (17.0)	
Brain stroke (%)	10 (38.5)	19 (35.8)	
Hemiplegia (%)	5 (19.2)	12 (22.6)	
Asthma (%)	5 (19.2)	5 (9.4)	
Others (%)	8 (30.7)	30 (56.6)	

BMI, body mass index; FIM, functional independence measure; CCI, Charlson comorbidity index; CKD, chronic kidney disease; COPD, chronic obstructive pulmonary disease; and MI, myocardial infarction. Others: malignancy, brain hemorrhage, liver diseases, kidney diseases, connective tissue diseases.

**Table 2:** Differences in the duration of hospitalization, mortality, and discharge rates.

Outcome	Intervention	Control	*p*-Value
Admission duration, days (interquartile)	61.50 (11.00, 480.00)	30.00 (4.00, 422.00)	0.008
Discharge from the hospital (%)	15 (57.7)	14 (26.4)	0.012
Death at the hospital (%)	9 (34.6)	38 (71.7)	0.003

**Table 3:** Diseases as reasons for patient admission.

No	Disease	*n*	Percentage	No	Disease	*n*	Percentage
1	Aspiration pneumonia	21	26.58%	5	Heart failure	2	2.53%
2	Bacterial pneumonia	20	25.32%	5	Brain stroke	2	2.53%
3	Urinary tract infection	13	16.47%	5	Dehydration	2	2.53%
4	Femoral neck fracture	3	3.80%	5	Appetite loss	2	2.53%
5	Acute respiratory distress syndrome	2	2.53%	6	Others	27	34.18%

## Data Availability

The datasets used and/or analyzed during the current study may be obtained from the corresponding author upon reasonable request.
